# Launching the Maternal and Neonatal section in the Primary Health Care and Family Medicine journal

**DOI:** 10.4102/phcfm.v16i1.4768

**Published:** 2024-11-15

**Authors:** Doreen K. Kaura

**Affiliations:** 1 Department of Nursing and Midwifery, Faculty of Medicine and Health Sciences, Stellenbosch University, Cape Town, South Africa

Maternal and Neonatal Health (MNH) is a critical area, particularly in Africa, where preventable deaths of women and neonates remain a significant public health concern.^1,2^ The introduction of a dedicated MNH section in the *African Journal of Primary Health Care and Family Medicine* (*PHCFM*) should promote research, inform policy and improve clinical practice. This editorial outlines the rationale behind this new section and the anticipated benefits for the journal and the wider medical community.

According to the World Health Organization, nearly 800 women die daily from avoidable causes associated with pregnancy and birth.^1,2^ Moreover, 2.4 million newborns died in 2019.^3^ Globally, over half of the annual 8.6 million maternal, perinatal, child, and adolescent deaths occur among stillborns (1.9 million) and neonates (2.3 million). These alarming statistics call for focused research and targeted policy interventions. *African Journal of Primary Health Care and Family Medicine* can provide a platform for African evidence to catalyse solutions that will enable Africa to improve health outcomes.^3^

Many countries in Africa have not significantly improved maternal mortality in line with sustainable development. To tackle maternal mortality, countries will require broader actions beyond biomedical issues and referral hospitals.^4,5^ Policymakers, need a renewed focus on strategies to address the underlying causes through a primary health care approach. Multisectoral policy and action as well as community empowerment will be essential to address the determinants of poor MNH.^5^ The bulk of MNH care falls within primary health care and district level health services, where family physicians can support midwives in improving care. The MNH section will promote the submission of high-quality research within primary health care and family medicine settings in Africa. It will provide a platform for innovative research that investigates interventions, practices, and policies for improved MNH.

Furthermore, the International Confederation of Midwives (ICM), through the Unifying Midwifery in Africa initiative, has been gearing towards collaborative research among midwives. These collaborations have demonstrated the potential for innovations in Maternal and Neonatal care in countries such as South Africa, Kenya, and Nigeria, which lead research outputs in this field.^6,7^ Building research capacity among African midwives and providing a scholarly platform for them to publish their work is a key focus for the PHCFM.

However, MNH also intersects with numerous disciplines, such as family medicine, obstetrics, paediatrics, neonatology, public and reproductive health. Furthermore, the complex relationships between gender equity, social determinants of health, and maternal outcomes highlight the need for interdisciplinary collaboration.^7,8^ The dedicated MNH section will create a platform for professionals from these various fields, to publish their research and share their perspectives. This interdisciplinary approach will lead to more inclusive solutions that direct the distinct needs of the African women and neonates.

The MNH section should improve health outcomes, influence policy, guidelines and best practice,^9^ extend the reach and utility of the journal, and enable research capacity building. Articles that provide evidence of preventive measures, early interventions, and enable evidence-based policies,^10,11^ will provide practitioners and policymakers with the strategies to save lives and improve the quality of care. Our focus is on primary health care and primary hospital settings with district health services. This body of evidence can drive systemic changes aimed at reducing MNH disparities across Africa.

The section will enhance the journal’s visibility and broaden its readership. The PHCFM has an open access and developmental philosophy to support early career and emerging researchers and to provide a unique platform for African scholarship and evidence. The PHCFM is the leading journal in its field in Africa and is rated 16 out of 56 family practice journals globally. With a SCOPUS Cite Score of 3.3, it provides all researchers in the field of MNH with the credibility and visibility they need for the dissemination of their work. We trust that PHCFM will help cultivate the next generation of leaders in MNH, fostering a vibrant and sustainable research community.^12^
